# Visual Exposure to Natural Environments Decreases Delay Discounting of Improved Air Quality

**DOI:** 10.3389/fpubh.2019.00308

**Published:** 2019-10-25

**Authors:** Meredith S. Berry, Meredith A. Repke, Lucian G. Conway

**Affiliations:** ^1^Human Behavioral Pharmacology and Decision-Making Laboratory, Department of Health Education and Behavior, University of Florida, Gainesville, FL, United States; ^2^Department of Psychology, University of Florida, Gainesville, FL, United States; ^3^Department of Psychology, University of Montana, Missoula, MT, United States

**Keywords:** delay discounting, intertemporal choice, natural vs. built environments, air quality, sustainability, behavioral economics, conservation psychology

## Abstract

Poor air quality contributes to nearly 7 million premature deaths annually and remains a major public health concern. In order to directly address the future of air quality and current emissions, some economists and policy makers have stressed adopting a “zero discount rate” (or lowest possible) to promote clean air quality now and in the future. A low discount rate is also associated with individual health behaviors (e.g., exercise and lower rate of substance abuse). But what influences the psychology of decision-making that is relevant to the discount rate of air quality and public health outcomes, and individual health? The present experiments evaluated differences in such decision-making (i.e., delay-discounting) in the context of improved air quality with visual exposure to natural vs. built environments. Results showed that individuals exposed to natural scenes discounted improved air quality less (i.e., made more future-oriented decisions, Experiment 1), and this may be related to expanded space perception (Experiment 2). These results are the first to suggest that delay discounting of air quality (or any environmental outcome), similar to money, is malleable, and can be influenced by exposure to natural relative to built environments. These findings have implications for influencing long-term, individual health, and environmentally relevant decision-making and improving individual and public health related outcomes such as air quality.

## Introduction

The World Health Organization (WHO) estimates that poor air quality resulting from emissions (e.g., from automobiles/industries/factories) contributes to nearly 7 million premature deaths worldwide each year (1). Further, the American Lung Association (ALA) estimates that over 50% of Americans breathe poor quality air that could lead to health problems. Improving air quality could drastically reduce the burden of stroke, heart disease, and lung cancer, as well as acute and chronic respiratory diseases, namely asthma (1). Air pollution is also a significant contributor to local and global biodiversity loss (2), and can interact with other anthropogenic influences such as climate change to impact the distribution of plant species, food availability, and reproductive success of wildlife species in polluted areas. Importantly, air quality does not constitute a single problem, but represents an array of threats to humans, public health, plants, and animals (1, 2).

In order to directly address the future of air quality and current emissions, some economists and policy makers have stressed adopting a “zero discount rate” (or the lowest possible) to promote clean air quality now and in the future [see Weitzman et al. (3) for discussion]. Delay discounting, or the discount rate, can be described as the degree to which outcomes in the future are devalued (4, 5). A zero discount rate implies that the future cannot be discounted relative to the present. In other words, to conserve clean air quality in the future citizens and policy makers must act in ways that drastically reduce negative anthropogenic influences on air quality *immediately* (e.g., substantially reduce emissions now from industry/factory/private car use), and thereby discount the future of air quality *less*. Behavior of society and individuals, however, shows that we discount the future at an alarmingly steep rate.

Why do we discount the future when steep discounting can be harmful for our health and the environment? In the case of both the environment and personal health, negative consequences may be relatively far in the future and probabilistic (e.g., climate change and lung cancer). When future consequences fail to influence present behavior, people act in ways that emphasize present bias (6). For example, it is convenient to drive a private car immediately despite increased emissions, rather than plan to take public transportation associated with an increased delay, but decreased overall emissions—a decrease that might improve air quality in the region they live in. Additionally, if many other people are also driving a private car, there may be less incentive to decrease one's own private car use. That is, individual emissions may seem minimally impactful compared to larger group emissions. Although many factors are at play in this example (e.g., immediate vs. delayed consequences, self vs. public health, and probability of experiencing an outcome), the basic tension of choice surrounding smaller sooner vs. larger later environmental outcomes is clear.

Although evidence suggests delay discounting is in part a function of individual differences [genetic influences (7); neurocognitive influences (8)] a distinct line of research has emerged focusing on the malleability of individual discount rates in laboratory settings—and subsequent changes in real world decision-making. This may represent implications for global reductions in delay discounting [e.g., drug use, overeating (9–13)]. Specifically, experimental manipulations have been designed to reduce or preclude the effects of delay on the value of money in order to facilitate a decrease in delay discounting. For example, Daniel et al. (10) showed that future episodic thought resulted in decreased delay discounting of money, and also less *ad libitum* energy consumption in obese individuals [see also (9); as well as (13) for examples of decreased delay discounting in monetary choices that correlated with decreased drug use].

This line of research would suggest that such methodologies used to decrease delay discounting of monetary outcomes [e.g., working memory training (14); visual exposure to nature (15–17); future episodic thought (13, 18); framing effects (19–21); for a review see Koffarnus et al. (22)], might also be used to decrease delay discounting of environmental outcomes more generally, and air quality specifically. This would lend theoretical evidence to targeting the same underlying cognitive and decision-making processes to enable decreased delay discounting of air quality (i.e., greater valuation of air quality in the future). We also targeted a potentially practical method to inform intervention to reduce delay discounting of air quality (e.g., increased exposure to natural spaces in cities or schools, incorporation of visual scenes of nature inside buildings).

Delay discounting of environmental outcomes and related measures is a burgeoning area of research (6, 23–26). A central discussion point is the degree that delay discounting processes operate in a similar fashion for far-reaching collective environmental decisions as they do for personal monetary decisions. Most research in the area is on personal monetary decisions. There are reasons to expect both fundamental differences [e.g., environmental decisions can be driven more by emotion than monetary decisions (27)] and fundamental similarities [e.g., strong positive correlations between money and environmental commodity discount rates (28)] between monetary and environmental decision-making processes. Such a discussion has theoretical and practical implications for the generality of delay discounting, associated factors influencing decision-making processes, and real world health and environmentally relevant behaviors [see Arbuthnot et al. (29) for discussion of the relation between delay of gratification and sustainability; see Hirsh et al. (30) for discussion of delay discounting as a psychological construct of sustainable behavior].

Other than a single study examining standard magnitude effects (31), no research to date has fused these specific areas of research to examine the malleability of discount rates in the context of environmental outcomes generally, or air quality specifically (i.e., at present comparisons are based solely on correlational measures). As such, it is currently unknown if the same manipulations designed to minimize effects of delay on monetary value would also minimize the effects of delay on air quality value (or the value of any environmental outcome). If delay discounting of air quality is malleable, this would offer vast implications for the ability of society to structure the environment to promote decreased delay discounting of air quality—and healthier long-term decision-making (i.e., a lower discount rate).

In the present experiments, we examined whether visual exposure to natural as opposed to built environments would result in decreased delay discounting of improved air quality (Experiment 1). We also examined potential underlying mechanisms driving this effect (Experiment 2), as has previously been shown with monetary outcomes (15–17). If delay discounting of improved air quality is decreased with visual exposure to natural as opposed to built environments, this would suggest (1) that how individuals value the future of air quality is malleable; (2) delay discounting of air quality can be reduced in a way that also reduces delay discounting of money, suggesting fundamentally similar, rather than fundamentally different underlying decision-making processes; and (3) the effects of decreasing delay discounting (i.e., increasing future valuation, increasing self-control) through visual exposure to natural environments is not domain specific (i.e., does not just apply to money), and may represent a generalizable and broad beneficial influence of exposure to natural environments on decision-making. We hypothesized that visual exposure to natural as opposed to built environments would decrease delay discounting of improved air quality.

## Experiment 1 Materials and Methods

### Participants

Sixty-six undergraduate students were recruited from an introductory psychology course and received course credit for participation. This study was carried out in accordance with the recommendations of University of Montana Institutional Review Board (IRB). The protocol was approved by the UM IRB. All subjects gave written informed consent in accordance with the Declaration of Helsinki.

### Setting and Apparatus

Participants were tested individually in a quiet experimental room containing a desk, chair, computer, and mouse. The room was well lit with overhead florescent lighting. Participants were not permitted to have personal belongings or cell phones on their person while participating in this experiment. Experimental manipulations and data recording were programmed using E-Prime 2.0^®^, and survey and demographic data were collected using Qualtrics^®^ (Provo, Utah). The monitor used was ~33 cm high, and 58 cm wide.

### Stimuli

The stimuli used in the present study have been used previously in delay discounting research, as well as attention restoration research [e.g., (15, 16, 32)]. Participants in the natural condition viewed photographs of nature (e.g., lakes, forests, and mountains), and participants in the built condition viewed photographs of built environments (e.g., cities, buildings, and roads). Each condition specific photograph contained primarily visual scenes from either natural or built scenes. In other words, nature scenes did not contain built aspects within the photograph and vice versa. The brightness of the photos were approximately equal across both natural and built photographs. For examples of the stimuli used, please see Berto (32) and Berry et al. (15).

### Procedure

Participants first read and signed informed consent. Participants were then seated in front of the computer and instructed to choose whichever option they preferred in the task, and that there were no “right” or “wrong” answers [similar to instructions provided by Odum et al. (33)]. Similar instructions were also provided on the screen, and led participants through the task. Participants used the mouse to progress through the experiment. Participants were assigned via block randomization to either the natural or built condition. Before the start of the delay discounting task (described below) and between each delay block, participants viewed either natural or built photographs. Photographs were displayed for 10-s each. Prior to the delay discounting task participants viewed 25 photographs, and between each delay block viewed five photographs (which were randomly selected from the original set of 25). With the exception of the differing photographs across natural or built conditions, all experimental procedures were identical.

In the delay-discounting task, participants made choices between experiencing hypothetical air quality improvements immediately or in the future. During the choice portion of the overall procedure, there was no limit on how much time the participants could take to select their choice in the delay discounting task. Participants read a hypothetical scenario that stated that the local county government was considering changes to its emissions policies (e.g., reducing pollutants of nearby factories) to test the effects of air quality on human health and local wildlife. These changes would result in a reduction of particulate output and pollutants in their area as measured by the Air Quality Index *(particulate matter, carbon monoxide, sulfur dioxide, nitrogen dioxide, and ground level ozone; 0* = *very good air quality to 500* = *hazardous air quality)* designated by the United States Environmental Protection Agency. As a result, substantial improvements in air quality would be experienced. The local government was considering making these changes for a number of days, after which time the air quality index would return to its former level, and the government was considering making these changes either immediately or sometime in the future. The Air Quality Index was selected as a means to describe air quality improvements to facilitate understanding of the hypothetical scenario for several reasons. First, it is an actual measure used by the U.S. Environmental Protection Agency. Second, it offers an accessible description of air quality, and finally many people in the U.S. are familiar with this measure. This hypothetical scenario was similar to the scenario presented by Hardisty and Weber (28).

For each trial in the adjusting amount delay discounting procedure employed in the present study, participants chose between an immediate smaller option, or a delayed larger option. Each choice screen read, “Would you rather experience [Y days of improved air quality] immediately,” or “Y days of improved air quality in [delay]?,” starting with values of 50 days immediately or 100 days after a delay on the first trial at each delay. Participants selected either the immediate (fewer days of improved air quality) or delayed outcome (more days of improved air quality) to progress, and the choices switched sides throughout the experiment. The mouse pointer was programmed to center after each selection. The immediate amount increased or decreased based on the participant's response as previously described [see Du et al. (34); Rodzon et al. (35) for a detailed descriptions of the titration procedure], so that the point at which the smaller sooner amount was equivalent to the larger later amount (i.e., the indifference point) was determined by the end of 10 trials at each delay. Delays tested were 1 day, 1 week, 1 month, 6 months, 1 year, 5 years, and 25 years in that order. Following all experimental procedures, basic demographic information was collected (e.g., age and sex).

### Data Analysis

To evaluate decision-making within the delay discounting task, two measures were assessed: Area Under the Curve (AUC) and *k* values. Area under the curve (AUC) was used to evaluate smaller sooner vs. larger later decision-making in the delay discounting air quality task, and as a comparison between decision-making across conditions. To calculate AUC the delays and indifference points at each delay (i.e., the point at which the smaller sooner and larger later amount was equivalent) were first normalized. Then the area underneath the discounting curve was calculated using the equation:

$$\begin{array}{l} (x2-x1)\;[(y1+y2)/2]\; \end{array}$$

where x2 and x1 represent successive delays and y1 and y2 represent the indifference points associated with those delays (36). These values were then summed, resulting in AUC calculations that range from 0 to 1, with lower numbers representing preference for smaller immediate outcomes (i.e., higher discount rates), and higher numbers representing preference for larger delayed outcomes (i.e., lower discount rates). A two-tailed Mann Whitney *U*-test was then used to test for potential differences in AUC across natural and built conditions. Values for AUC were not normally distributed, so statistical analyses presented do not assume Gaussian distributions (i.e., a Mann Whitney *U*-test was used). The second measure of discounting, *k* values (which represent degree or “rate” of discounting), were examined using the widely employed simple hyperbola (4):

$$\begin{array}{l} V=A/(1+kD)\; \end{array}$$

where V is the subjective value of the reward, D is the delay to receipt of the reward, and *k* is the degree to which the value of the reward decreases with delay. The values of A and D are predetermined based on the value used within the research context [e.g., if the delayed reward used is 100 dollars (or days of improved air quality), then the numerator is 100]. *k* values are then compared across conditions (using a Mann Whitney *U*-test to account for non-normal distributions).

## Experiment 1 Results

Of the 66 participants, 57% were female. The mean age was 23.62 years (SD = 7.79). Chi square (sex, race) and a *t*-test (age) comparing demographics across natural and built conditions indicated no significant differences across groups for these variables (sex, *p* = 0.451; race, *p* = 1.00; age, *p* = 0.633).

**Figure 1:**
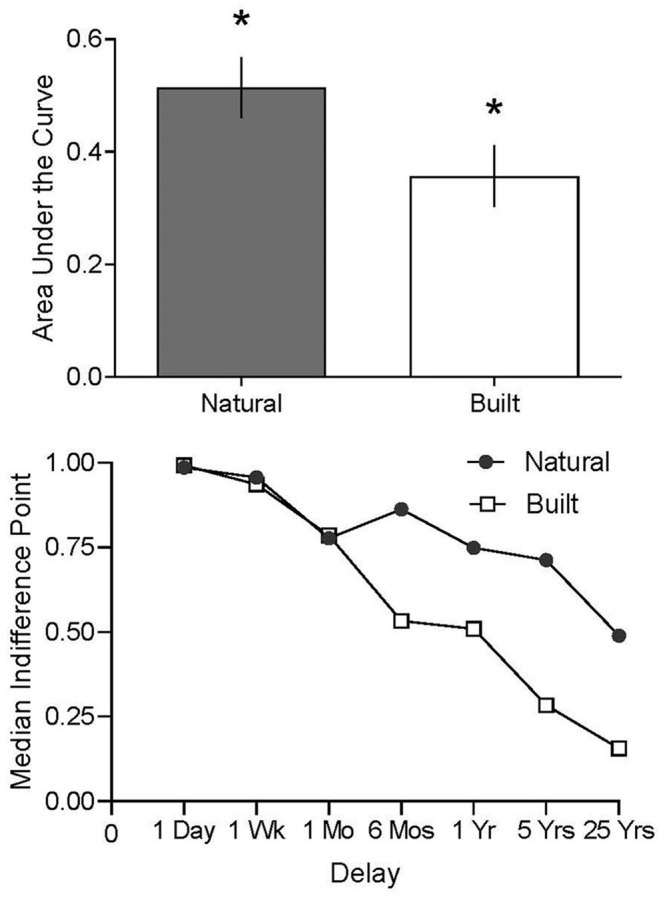
The top panel displays mean AUC for the natural (filled bar) and built (open bar) conditions. Vertical lines represent the standard error of the mean. Asterisks represent statistically significant differences in AUC between the natural and built conditions. The bottom panel displays median indifference points across natural (filled circle) and built (open square) conditions as a function of delay. Delays spaced equidistantly to facilitate inspection of all values (with x-axis labels showing specific delays assessed).

Figure 1 displays the AUC for the natural and built conditions. Participants who viewed scenes of natural as opposed to built environments chose to experience more days of improved air quality with a delay, to fewer days of improved air quality immediately (i.e., higher AUC values; Natural Condition Mean = 0.514, SE = 0.054; Built Condition Mean = 0.357, SE = 0.055). A two-tailed test confirmed these visual assessments showing significant differences in AUC across natural and built conditions (Mann-Whitney *U*-test; *U* = 383.5, *p* = 0.041). Similar results were revealed when testing *k* parameter values across natural and built conditions (Mann-Whitney *U*-test; *U* = 335, *p* = 0.041).

## Experiment 1 Discussion

Experiment 1 findings showed that those who viewed photos of nature discounted air quality less than those who viewed photos of built environments, suggesting that delay discounting of air quality is malleable. It is untested, however, if similar mechanisms underlie these behavioral effects as has been shown with monetary outcomes. Specifically, time and space perception appear to be related to reduced delay discounting of monetary outcomes with visual exposure to nature. To determine if these mechanisms also underlie decisions related to improved air quality with visual exposure to natural vs. built environments, we also examined time and space perception, as time/space perception has been predicted to influence both delay discounting and differentially affected by viewing natural vs. built environments (16, 37). We predicted that both time and space perception would be expanded with visual exposure to natural as opposed to built environments.

## Experiment 2 Materials and Methods

Forty-two undergraduate students were recruited from an introductory psychology course and received course credit for participation. This study was carried out in accordance with the recommendations of University of Montana Institutional Review Board (IRB). The protocol was approved by the UM IRB. All subjects gave written informed consent in accordance with the Declaration of Helsinki. The setting and apparatus, stimuli, procedure and data analysis for the discounting data were identical to the methods used in Experiment 1.

Following the completion of the discounting portion of the study, participants then answered several questions to assess time and space perception. Specifically, “How many minutes would you estimate have elapsed since signing the informed consent?,” and “Picture yourself immersed in the images you saw on the screen throughout the study. On a scale of 1 (constricted)−10 (expanded), how does the space feel around you when you do this?” Finally, basic demographic information was collected.

## Experiment 2 Results

Of the 42 participants, 71% were female. The mean age was 20.48 years (SD = 3.46). Chi square (sex, race) and a *t*-test (age) comparing demographics across natural and built conditions indicated no significant differences across groups for these variables (sex, *p* = 0.303; race, *p* = 0.687; age, *p* = 0.347).

**Figure 2:**
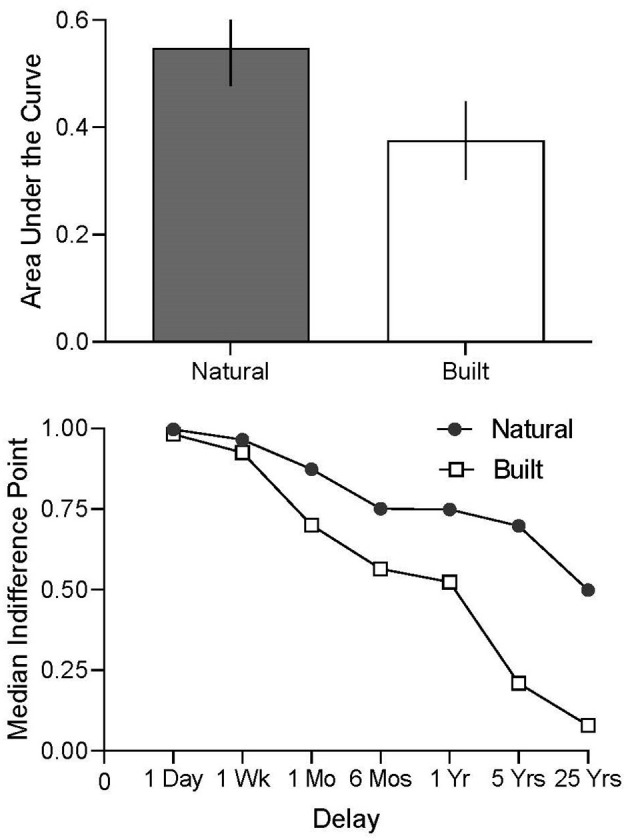
The top panel displays mean AUC for the natural (filled bar) and built (open bar) conditions. Vertical lines represent the standard error of the mean. The bottom panel displays median indifference points across natural (filled circle) and built (open square) conditions as a function of delay. Delays spaced equidistantly to facilitate inspection of all values (with x-axis labels showing specific delays assessed).

Figure 2 displays the AUC for the natural and built conditions for Experiment 2. Participants who viewed scenes of natural as opposed to built environments chose to experience more days of improved air quality with a delay, to fewer days of improved air quality immediately (i.e., higher AUC values; Natural Condition Mean = 0.548, SE = 0.071; Built Condition Mean = 0.376, SE = 0.073), although these differences only trended toward standard levels of significance (Mann-Whitney *U*-test; *U* = 151.5, *p* = 0.103). Similar results were observed when testing *k* parameter values across natural and built conditions (Mann-Whitney *U*-test; *U* = 113.5, *p* = 0.065). Despite the lack of significant differences observed across the natural and built conditions, the effect was in the predicted direction, similar to the outcomes observed in Experiment 1.

Participants estimated a similar passage of time in minutes in both the natural and built conditions [Natural Condition Mean = 20.0, SE = 1.8; Built Condition Mean = 21.1, SE = 1.5, unpaired *t*-test, *t*_(40)_ = 0.47, *p* = 0.64]. Participants in the natural condition, however, reported significantly expanded space perception relative to those in the built condition [Natural Condition Mean = 5.6, SE = 0.50; Built Condition Mean, M = 3.8, SE = 0.59; *t*_(40)_ = 2.26, *p* = 0.03].

## Experiment 2 Discussion

Experiment 2 results showed a trend toward reduced delay discounting of air quality outcomes with viewing natural as opposed to built environments. This trend however, was not statistically significant. There is also some indication that space perception is expanded with exposure to natural as opposed to built environments, but less so for time, partially replicating previous results (37).

## General Discussion

First and foremost, these results offer the first direct evidence that viewing nature scenes can cause people to be future-oriented in their approach to air quality decisions: When people viewed nature scenes, they were more likely to trade bad days now for a better future. This finding has multiple novel implications: (1) Delay discounting of air quality is malleable acutely in an experimental context, (2) processes underlying delay discounting in environmental vs. monetary outcomes may be similar, and (3) although more research is needed, there are potential broad benefits of exposure to natural environments for human decision-making that emphasizes future rather than present outcomes, (4) expanded space perception may contribute to these outcomes, however results in this domain remain preliminary. We discuss each of these in turn.

### Delay Discounting of Air Quality Is Malleable

Those who viewed photographs of natural as opposed to built scenes chose to experience more days of improved air quality in the future, to fewer days of improved air quality immediately. These results are the first to show that delay discounting in the context of environmental outcomes generally, and air quality more specifically, is responsive to acute experimental manipulations of natural or built photograph exposure. In particular, environments in which we spend our time appear to influence how individuals value future air quality. Some economists have stressed adopting the lowest possible discount rate associated with environmental outcomes to promote a healthier future (i.e., cleaner air). More access and increased exposure to natural/green spaces may facilitate individuals and society to place greater value on future rather than present air quality (thereby decreasing discount rates), and potentially other environmental outcomes.

### Some mechanisms Underlying Delay Discounting of Environmental and Monetary Outcomes May Be Similar

Although little direct data exist, there has nonetheless been some discussion concerning exactly how similar the decision-making processes surrounding monetary and environmental discounting are to each other. For example, Gattig and Hendrickx (27) have suggested that decisions of risk and time related to the environment are driven more by emotion than decisions regarding money. Hardisty and Weber (28), however, have proposed that decisions for environmental and monetary outcomes are driven by similar underlying processes, as evidenced by the positive correlations between delay discounting of environmental and monetary commodities. Although necessary to establish similarities in decision-making processes [see Green and Myerson (38) for discussion of similarities and differences between delay and probability discounting], no previous experiments have tested whether the same manipulations designed to minimize the effect of delay in the context of monetary outcomes, might also minimize the effects of delay in the context of air quality outcomes (or other environmental outcomes). These results are the first to provide experimental evidence to the discussion—showing that decreased delay discounting of improved air quality also results from visual exposure to natural as opposed to built environments, as has been shown with delay discounting of money (15–17). These data lend support to the hypotheses that decision-making processes underlying environmental and monetary outcomes may be more similar than different. More research is needed to better understand similarities and differences between monetary and environmental decision-making.

What might account for the similarities? The expansion of time and space perception have been isolated as a potential mechanisms driving decreases in delay discounting with visual exposure to natural environments (16, 37), as time and space perception and temporal attention and delay discounting are related [e.g., (21, 37, 39)]. Expansion of space perception but not time perception with exposure to natural environments was demonstrated in Experiment 2 of the present study, lending partial evidence to these mechanisms. Berry, Repke, and other colleagues have speculated that expansion of time and/or space perception induced by visual exposure to natural environments may facilitate bridging the gap between current choices and future consequences. In addition to differences in time perception, previous research has shown differences in mood (40, 41), as well as differences in attention (32) and eye movements [e.g., saccades and fixations (42)] that are associated with tracking scenes of natural vs. built environments, each of which also influence delay discounting [e.g., attention (43); mood (44)]. All of these factors might apply equally across all domains that involve some form of delay discounting, including monetary and air quality decisions. More research is needed to squarely identify the mechanistic drivers of decreased delay discounting with exposure to natural as opposed to built environments [for other examples of differences in cognition and behavior with exposure to natural environments see Rutiku et al. (45) for differences in electro encephalography responses and cortical alpha activity with visual exposure to task irrelevant natural or built environments; as well as (46) for increases in creative problem solving with immersion in natural environments].

### Practical Implications of Exposure to Nature on Air Quality

Although more research is needed to understand the mechanistic underpinnings, these findings have initial implications for how society might consider structuring environments to promote sustainable decision-making geared toward clean air in the future. Future research might identify if, for example, cities might be structured with more natural and green spaces to promote long term, future oriented decision-making that could subsequently benefit the environment (e.g., walking or taking public transportation rather than driving a private car). Increased implementation of natural spaces within cities, workplaces, and schools, as well as natural scenes within buildings could increase happiness and well-being (40), and also outcomes related to short-term impulsive decision-making [e.g., (15, 16)], and long-term environmental health (the present study).

This research combined with previous studies suggests that beyond frequent findings of increased happiness [e.g., (40)], improved attention [e.g., (32)], and reduced stress [e.g., (41)], exposure to natural as opposed to built environments also influences *decision-making* in the context of delay discounting, an important predictor of *real world behavior* [see also (47); for an example of increased cooperation with exposure to nature]. Reductions in delay discounting of air quality in real world situations will be necessary to address the effects of current and future emissions for our health and the health of our ecosystems (48). Importantly, exposure to natural as opposed to built environments may facilitate a beneficial and reciprocal interaction between humans and our ecosystems, with focus toward maximizing future ecosystem health, and therefore human health. Although delay discounting as a process (specifically a low discounting rate) has been considered as a potential fundamental aspect needed to engage in long term sustainable behaviors (30), more research is needed to understand the association between rate of delay discounting and environmentally relevant behaviors.

Several limitations exist. Our air quality scenarios are necessarily hypothetical. Research has shown, however, that decisions about hypothetical outcomes in a laboratory context are associated with real world behavior and choices involving delays (49, 50). By using a hypothetical scenario, however, we are able to show that air quality preferences are systematically influenced by delay, and the present procedure may represent the best way to study these relations under controlled conditions. Further, actual decision-making should be tested in future studies to more broadly address the influence of nature exposure on the value of air quality. Willingness to pay questions or scenarios in which individuals pay for improved air quality would offer increased breadth to the present findings. An additional limitation was the homogenous undergraduate student sample. This sample limits the generality of the findings. Future studies should examine these questions in older adult populations, as older adults tend to be involved in decision-making processes more than young people. Simultaneously, given improved air quality may be less likely to occur in the lifetime of an older adult, this may lead to higher discount rates. It is also unknown how an individual's experience with fluctuating air quality might contribute to the present findings.

It is also unknown how long such effects last, or exactly the “dose” of nature that is required to induce such effects. It should also be noted that the content of the photos used, such as lakes and forests, may not be representative of many natural environments and spaces. The same is true for the built environment photographs used in the present study. A large proportion of humans spend time in a combination of natural and built environments, and the current photos captured only one environment or the other. Additional studies parametrically incorporating both natural and built content within the photographs will help increase generalizability of these findings. Although the purpose of the present study was to compare the effects of natural vs. built environments on discounting of improved air quality, it should be noted that no neutral condition was used for further comparison. However, previous research (15) has found a neutral geometric condition to yield no differences in degree of money delay discounting when compared to the built condition. Future research should determine if visual exposure to natural, built, or neutral photographs yield differences in discounting of improved air quality.

Additionally, although the present studies offer some clues as to mechanistic underpinnings of the observed effects, future fully-powered studies should examine underlying mechanisms, as research in this area remains extremely limited. Lastly, it is unlikely that an individual's behavior would be influenced by exposure to natural environments if there is little to no attention paid to such surroundings (i.e., benefits are unlikely to be gleaned from surrounding nature if an individual is engrossed in their iPhone). Despite these limitations, these data are the first to reveal promising outcomes in the context of human decision-making with exposure to natural environments that might ultimately inform conservation of our present and future local and global air quality and improvement in public health.

## Data Availability Statement

All datasets generated for this study are included in the manuscript/supplementary files.

## Ethics Statement

This study was carried out in accordance with the recommendations of University of Montana Institutional Review Board (IRB). The protocol was approved by the UM IRB. All subjects gave written informed consent in accordance with the Declaration of Helsinki.

## Author Contributions

MB contributed to the conceptualization, data collection, formal analysis, methodology and writing the original draft, and review and editing of the manuscript. MR contributed to the conceptualization, data collection and analysis, and review and editing of the manuscript draft. LC contributed space and materials and review and editing of the manuscript draft.

### Conflict of Interest

The authors declare that the research was conducted in the absence of any commercial or financial relationships that could be construed as a potential conflict of interest.

## References

[1] World Health Organization 7 Million Premature Deaths Annually Linked to Air Pollution. (2017). Retrieved from: http://www.who.int/mediacentre/news/releases/2014/air-pollution/en/ (accessed December 2, 2016).

[2] Dudley N Stolton S Air Pollution and Biodiversity: A Review. Bristol: Montpelier (1996).

[3] Weitzman ML Why the far-distant future should be discounted at its lowest possible rate. J Environ Econ Manage. (1998) 36:201 10.1006/jeem.1998.1052

[4] Mazur JE An adjusting procedure for studying delayed reinforcement. In: Commons ML Mazur JE Nevin JA Rachlin H editors. Quantitative Analyses of Behavior, Vol. 5, The Effect of Delay and of Intervening Events on Reinforcement Value. Hillsdale, NJ: Earlbaum (1987). p. 55–73.

[5] Rachlin H. Notes on discounting. J Exp Anal Behav. (2006) 85:425. 10.1901/jeab.2006.85-05 16776060 PMC1459845

[6] Hepburn C Duncan S Papachristodoulou A Behavioural economics, hyperbolic discounting and environmental policy. Environ Resour Econ. (2010) 46:189 10.1007/s10640-010-9354-9

[7] Anokhin A Golosheykin S Grant J Heath A. Heritability of delay discounting in adolescence: a longitudinal twin study. Behav Genet. (2011) 41:175. 10.1007/s10519-010-9384-7 20700643 PMC3036802

[8] McClure SM Laibson DI Loewenstein G Cohen JD. Separate neural systems value immediate and delayed monetary rewards. Science. (2004) 306:503. 10.1126/science.1100907 15486304

[9] Black AC Rosen MI. A money management-based substance use treatment increases valuation of future rewards. Addict Behav. (2011) 36:125. 10.1016/j.addbeh.2010.08.014 20826055 PMC2981645

[10] Daniel TO Stanton CM Epstein LH. The future is now: reducing impulsivity and energy intake using episodic future thinking. Psychol Sci. (2013) 24:2339. 10.1177/0956797613488780 24022653 PMC4049444

[11] Odum AL. Delay discounting: I'm a *k*, you're a *k*. J Exp Anal Behav. (2011) 96:427. 10.1901/jeab.2011.96-423 22084499 PMC3213005

[12] Odum AL. Delay discounting: trait variable? Behav Process. (2011) 87:1–9. 10.1016/j.beproc.2011.02.007 21385637 PMC3266171

[13] Stein JS Wilson AG Koffarnus MN Daniel TO Epstein LH Bickel WK. Unstuck in time: episodic future thinking reduces delay discounting and cigarette smoking. Psychopharmacology. (2016) 233:3771–8. 10.1007/s00213-016-4410-y 27553824 PMC9812225

[14] Bickel WK Yi R Landes RD Hill PF Baxter C. Remember the future: working memory training decreases delay discounting among stimulant addicts. Biol Psychiatry. (2011) 69:260. 10.1016/j.biopsych.2010.08.017 20965498 PMC3015021

[15] Berry MS Sweeney MM Morath J Odum AL Jordan KE. The nature of impulsivity: visual exposure to natural environments decreases impulsive decision-making in a delay discounting task. PLoS ONE. (2014) 9:e97915. 10.1371/journal.pone.0097915 24841421 PMC4026519

[16] Berry MS Repke MA Nickerson NP Conway LG III Odum AL Jordan KE. Making time for nature: visual exposure to natural environments lengthens subjective time perception and reduces impulsivity. PLoS ONE. (2015) 10:e0141030. 10.1371/journal.pone.0141030 26558610 PMC4641682

[17] van der Wal AJ Schade HM Krabbendam L van Vugt M. Do natural landscapes reduce future discounting in humans? Proc Biol Sci R Soc. (2013) 280:20132295. 10.1098/rspb.2013.2295 24197412 PMC3826228

[18] Peters J Büchel C. Episodic future thinking reduces reward delay discounting through an enhancement of prefrontal-mediotemporal interactions. Neuron. (2010) 66:138. 10.1016/j.neuron.2010.03.026 20399735

[19] DeHart WB Odum AL. The effects of the framing of time on delay discounting. J Exp Anal Behav. (2015) 103:10. 10.1002/jeab.125 25524395 PMC5661958

[20] LeBoeuf RA Discount rates for time versus dates: the sensitivity of discounting to time-interval description. J Market Res. (2006) 43:59 10.1509/jmkr.43.1.59

[21] Radu PT Yi R Bickel WK Gross JJ McClure SM. A mechanism for reducing delay discounting by altering temporal attention. J Exp Anal Behav. (2011) 96:363. 10.1901/jeab.2011.96-363 22084496 PMC3213002

[22] Koffarnus MN Jarmolowicz DP Mueller ET Bickel WK. Changing delay discounting in the light of the competing neurobehavioral decision systems theory: a review. J Exp Anal Behav. (2013) 99:32. 10.1002/jeab.2 23344987 PMC3917566

[23] Johnson AE Saunders DK Time preferences and the management of coral reef fisheries. Ecol Econ. (2014) 100:130 10.1016/j.ecolecon.2014.01.004

[24] Kaplan B Reed D McKerchar T Using a visual analogue scale to assess delay, social, and probability discounting of an environmental loss. Psychol Rec. (2014) 64:261 10.1007/s40732-014-0041-z

[25] Meyer A Intertemporal valuation of river restoration. Environ Resour Econ. (2013) 54:41 10.1007/s10640-012-9580-4

[26] Viscusi WK Huber J Bell J The economic value of water quality. Environ Resour Econ. (2008) 41:169 10.1007/s10640-007-9186-4

[27] Gattig A Hendrickx L Judgmental discounting and environmental risk perception: Dimensional similarities, domain differences, and implications for sustainability. J Soc Issues. (2007) 63:21 10.1111/j.1540-4560.2007.00494.x

[28] Hardisty DJ Weber EU. Discounting future green: money versus the environment. J Exp Psychol Gen. (2009) 138:329. 10.1037/a0016433 19653793

[29] Arbuthnott KD Taking the long view: environmental sustainability and delay of gratification. Anal Soc Issues Public Policy. (2010) 10:4 10.1111/j.1530-2415.2009.01196.x

[30] Hirsh JL Costello MS Fuqua RW Analysis of delay discounting as a psychological measure of sustainable behavior. Behav Soc Issues. (2015) 24:187–202. 10.5210/bsi.v24i0.5906

[31] Berry MS Friedel JE DeHart WB Mahamane S Jordan KE Odum AL. The value of clean air: comparing discounting of delayed air quality and money across magnitudes. Psychol Rec. (2017) 67:137–48. 10.1007/s40732-017-0233-4 29606776 PMC5877464

[32] Berto R Exposure to restorative environments helps restore attentional capacity. J Environ Psychol. (2005) 25:249 10.1016/j.jenvp.2005.07.001

[33] Odum AL Baumann AAL Rimington DD. Discounting of delayed hypothetical money and food: effects of amount. Behav Process. (2006) 73:278. 10.1016/j.beproc.2006.06.008 16926071

[34] Du W Green L Myerson J Cross-cultural comparisons of discounting delayed and probabilistic rewards. Psychol Rec. (2002) 52:479 10.1007/BF03395199

[35] Rodzon K Berry MS Odum AL. Within-subject comparison of degree of delay discounting using titrating and fixed sequence procedures. Behav Process. (2011) 86:164. 10.1016/j.beproc.2010.09.007 20932882 PMC3919556

[36] Myerson J Green L Warusawitharana M. Area under the curve as a measure of discounting. J Exp Anal Behav. (2001) 76:235. 10.1901/jeab.2001.76-235 11599641 PMC1284836

[37] Repke MA Berry MS Conway LG III Metcalf A Hensen RM Phelan C. How does nature exposure make people healthier?: evidence for the role of impulsivity and expanded space perception. PLoS ONE. (2018) 13:e0202246. 10.1371/journal.pone.0202246 30133499 PMC6104990

[38] Green L Myerson J. A discounting framework for choice with delayed and probabilistic rewards. Psychol Bull. (2004) 130:769. 10.1037/0033-2909.130.5.769 15367080 PMC1382186

[39] Baumann AA Odum AL. Impulsivity, risk taking, and timing. Behav Process. (2012) 90:408. 10.1016/j.beproc.2012.04.005 22542458 PMC3897391

[40] White MP Alcock I Wheeler BW Depledge MH. Would you be happier living in a greener urban area? A fixed-effects analysis of panel data. Psychol Sci. (2013) 24:920. 10.1177/0956797612464659 23613211

[41] Ulrich RS Natural versus urban scenes: some psychophysiological effects. Environ Behav. (1981) 13:523 10.1177/0013916581135001

[42] Berto R Massaccesi S Pasini M Do eye movements measured across high and low fascination photographs differ? Addressing kaplan's fascination hypothesis. J Environ Psychol. (2008) 28:185 10.1016/j.jenvp.2007.11.004

[43] Barkley RA Edwards G Laneri M Fletcher K Metevia L. Executive functioning, temporal discounting, and sense of time in adolescents with attention deficit hyperactivity disorder (ADHD) and oppositional defiant disorder (ODD). J Abnorm Child Psychol. (2001) 29:541. 10.1023/A:1012233310098 11761287

[44] Hirsh JB Guindon A Morisano D Peterson JB. Positive mood effects on delay discounting. Emotion. (2010) 10:717. 10.1037/a0019466 21038955

[45] Rutiku R Einberg A Imanaka K Bachmann T. The effect of task-irrelevant visual backgrounds on human transcranial magnetic stimulation-evoked electroencephalography responses and cortical alpha activity. Eur J Neurosci. (2013) 38:3768. 10.1111/ejn.12374 24118584

[46] Ann Atchley R Strayer DL Atchley P Creativity in the wild: Improving creative reasoning through immersion in natural settings. PLoS ONE. (2012) 7:e51474 10.1371/journal.pone.0051474 23251547 PMC3520840

[47] Zelenski JM Dopko RL Capaldi CA Cooperation is in our nature: nature exposure may promote cooperative and environmentally sustainable behavior. J Environ Psychol. (2015) 42:24 10.1016/j.jenvp.2015.01.005

[48] Berry MS Nickerson NP Odum AL. Delay discounting as an index of sustainable behavior: devaluation of future air quality and implications for public health. Int J Environ Res Public Health. (2017) 14:997. 10.3390/ijerph14090997 28862671 PMC5615534

[49] Johnson MW Bickel WK. An algorithm for identifying nonsystematic delay-discounting data. Exp Clin Psychopharmacol. (2008) 16:264. 10.1037/1064-1297.16.3.264 18540786 PMC2765051

[50] Lagorio CH Madden GJ. Delay discounting of real and hypothetical rewards III: Steady-state assessments, forced-choice trials, and all real rewards. Behav Process. (2005) 69:173. 10.1016/j.beproc.2005.02.003 15845306

